# Beth Levine’s Legacy: From the Discovery of BECN1 to Therapies. A Mentees’ Perspective

**DOI:** 10.3389/fcell.2022.891332

**Published:** 2022-06-27

**Authors:** Zhenyi An, Wei-Chung Chiang, Álvaro F. Fernández, Luis H. Franco, CongCong He, Shu-Yi Huang, Eunmyong Lee, Yang Liu, Salwa Sebti, Sanae Shoji-Kawata, Shyam Sirasanagandla, Richard C. Wang, Yongjie Wei, Yuting Zhao, Silvia Vega-Rubin-de-Celis

**Affiliations:** ^1^ Broad Institute of MIT and Harvard, Cambridge, MA, United States; ^2^ Institute of Biochemistry and Molecular Biology, College of Life Sciences, National Yang Ming Chiao Tung University, Taipei, Taiwan; ^3^ Departamento de Bioquímica y Biología Molecular, Instituto Universitario de Oncología (IUOPA), Universidad de Oviedo, Instituto de Investigación Sanitaria del Principado de Asturias (ISPA), Oviedo, Spain; ^4^ Departamento de Bioquímica e Imunologia, Instituto de Ciências Biológicas, Universidade Federal de Minas Gerais, Belo Horizonte, Brazil; ^5^ Department of Cell and Developmental Biology, Feinberg School of Medicine, Northwestern University, Chicago, IL, United States; ^6^ Department of Medical Research, National Taiwan University Hospital, Taipei, Taiwan; ^7^ InnoCure Therapeutics Inc., Gyeonggi-do, South Korea; ^8^ Department of Integrative Biology and Pharmacology, McGovern Medical School, University of Texas Health Science Center, Houston, TX, United States; ^9^ Department of Internal Medicine, University of Texas Southwestern Medical Center, Dallas, TX, United States; ^10^ Department of Pharmacy, Nishi Nara Central Hospital, Nara, Japan; ^11^ St Jude Children’s Research Hospital, Memphis, TN, United States; ^12^ Department of Dermatology, University of Texas Southwestern Medical Center, Dallas, TX, United States; ^13^ Cancer Research Institute, Guangzhou Medical University, Guangzhou, China; ^14^ Institute of Future Agriculture, Northwest A&F University, Yangling, China; ^15^ Institute for Cell Biology (Cancer Research), Essen University Hospital, University of Duisburg-Essen, Essen, Germany

**Keywords:** BECN1, macroautophagy, selective autophagy, cancer, aging, metabolism, infectious diseases, Beth Levine

## Abstract

With great sadness, the scientific community received the news of the loss of Beth Levine on 15 June 2020. Dr. Levine was a pioneer in the autophagy field and work in her lab led not only to a better understanding of the molecular mechanisms regulating the pathway, but also its implications in multiple physiological and pathological conditions, including its role in development, host defense, tumorigenesis, aging or metabolism. This review does not aim to provide a comprehensive view of autophagy, but rather an outline of some of the discoveries made by the group of Beth Levine, from the perspective of some of her own mentees, hoping to honor her legacy in science.

## 1 Introduction

It all started with an essential paper, published by the Levine lab, reporting the description of BECN1 as a BCL2-binding protein connecting two essential pathways, autophagy and apoptosis (Liang et al., 1998). The ultimate goal of the autophagic pathway is the influx of different types of cargo into the lysosome for its degradation (Galluzzi et al., 2017). This delivery can be achieved by different means (Parzych and Klionsky, 2014). For example, the lysosome itself can engulf small substrates by the invagination of its membrane, in a process termed “microautophagy” (Mijaljica et al., 2011). Additionally, some proteins that show a KFERQ motif can be recognized by LAMP2A and chaperone Hsc70 (HSPA8) so they are specifically internalized into the lysosome, resulting in “chaperone-mediated autophagy” (or CMA) (Kaushik and Cuervo, 2018). Finally, unspecific bulk portions of the cytoplasm or big structures, like organelles or pathogens, can be sequestered by a double-membrane vesicle called “autophagosome,” which then fuses with a lysosome to form an “autolysosome” where the final degradation occurs. This specific way of cargo delivery is called “macroautophagy” or simply “autophagy,” and it involves the coordinated activity of a complex array of proteins known as the autophagy core machinery (Kawabata and Yoshimori, 2020). This group of autophagy effectors orchestrates different steps of the route, from the regulation of its activation and the initiation of the autophagosome formation (where BECN1 is crucial) to the completion of this structure, its transport, and its fusion with the lysosome. Back in 1999, the Levine laboratory discovered that BECN1 was the mammalian ortholog of the yeast Vps30/Atg6, providing evidence of a rescued autophagy function both in Atg6-deficient yeast and in breast cancer cells (MCF7) expressing low BECN1 levels (Liang et al., 1999). Similar to Atg6, BECN1 was then found to be a part of Class III PI3K lipid kinase complexes (Kihara et al., 2001), including Class III PI3K-C1 complex (containing BECN1, VPS34, VPS15 and ATG14) and Class III PI3K-C2 complex (containing BECN1, VPS34, VPS15 and UVRAG) (Sun et al., 2008; Matsunaga et al., 2009; Zhong et al., 2009), which mediate autophagosome formation and maturation, respectively (Levine et al., 2015).

**FIGURE 1:**
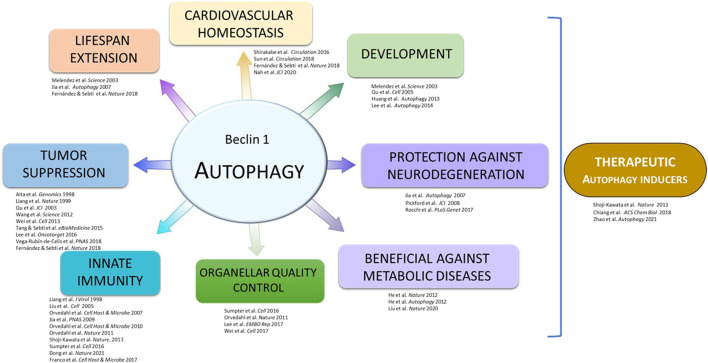
Outline of discoveries from the Beth Levine laboratory.

One key mechanism that regulates the initiation of autophagy is the activation of the Class III PI3K-C1 complex. In a crucial work published in 2005, the Levine lab demonstrated that BCL2 interaction with BECN1 negatively regulates autophagy induction (Pattingre al., 2005). In basal conditions, BCL2 binds to the BH3 domain of BECN1 and limits the activation of the Class III PI3K-C1 complex (Pattingre et al., 2005). However, the BECN1/BCL2 interaction is disrupted in response to stress stimuli such as nutrient starvation, leading to upregulation of autophagy. Furthermore, introducing of a Phe-to-Ala point mutation in amino acid 123 (F123A) in the BH3 domain of human BECN1 was shown to completely disrupt BECN1 binding to BCL2 *in vitro,* and lead to an increase in BECN1- Class III PI3K complex lipid kinase activity and induction of autophagy (Pattingre et al., 2005; Sinha et al., 2008; Wei et al., 2008). These remain seminal discoveries in the autophagy field that set up the foundation of a series of essential findings for understanding the role of autophagy in health and disease (Figure 1).

## 2 Autophagy in Overall Health and Development

### 2.1 Autophagy in Aging

One of the main lines of research at Beth Levine’s lab was the link between BECN1 (and autophagy) and longevity. Several studies have described that autophagy declines during aging while its stimulation (either by physiological or pharmacological inducers) extends lifespan in different animal models (Hansen et al., 2018; Kaushik et al., 2021). In this regard, Beth Levine showed that bec-1 (the BECN1 orthologue of *Caenorhabditis elegans*) mediates the effect of insulin signaling and dietary restriction on the regulation of nematode lifespan (Melendez et al., 2003; Jia and Levine, 2007). Autophagy activation is an important survival mechanism under stress conditions, and in *C. elegans*, the larvae can enter an alternative stage of development called dauer if the environmental stress is too high (no food, high temperature, etc.). The dauer stage not only enhances survival under harsh conditions but also extends lifespan. Meléndez et al. evaluated the effect of autophagy inhibition in worms with a mutation in the daf-2 gene, which encodes the insulin-like growth factor 1 (IGF-1) receptor (Melendez et al., 2003). The temperature-sensitive mutant daf-2(e1370) forms dauer constitutively at high temperature and has a prolonged lifespan at low temperature. Careful characterization revealed that the bec-1(RNAi) animals form abnormal dauers die within a few days, and the survivors lose the ability to reproduce. Moreover, bec-1(RNAi) greatly reduced the lifespan of daf-2(e1370) under low temperature, indicating that autophagy is required for the extended lifespan in this mutant. Autophagic activity markedly increased in daf-2 (e1370) dauer, and RNAi depletion of several other autophagy genes results in dauer formation defects and abnormal autophagosome patterns. This study confirmed the conserved roles of *C. elegans* autophagy orthologs and established the requirement of autophagy in dauer formation and lifespan extension via modulating the insulin signaling pathway.

Dietary restriction, another mechanism that extends lifespan, is also an autophagy modulator (Jia et al., 2007). Using a *C. elegans* model of mutant eat-2(ad1113), which has a dysfunctional pharynx resulting in reduced food intake, bec-1 and atg-7 were shown to be required for the longevity phenotype on this mutant. These findings indicate that autophagy does not only mediate longevity via the insulin signaling pathway but also mediates the effect of dietary restriction to promote longevity.

Even more importantly, the recent description of a murine model with a gain-of-function point mutation in Becn1 has further proved that BECN1 activity is also linked to longevity and health preservation in mammals (Fernandez et al., 2018). Specifically, mice expressing F121A mutation on Becn1 (which frees this protein from its negative regulator BCL2) not only showed enhanced autophagy levels that were sustained into their adulthood in different tissues but also lifespan extension in both females and males, living 10% longer than wild-type mice. Furthermore, this increase in longevity was associated with a marked improvement of their health, as these animals displayed decreased age-related alterations in heart and kidney, decreased incidence of age-related spontaneous tumors, and protection against neurodegeneration (Rocchi et al., 2017; Fernandez et al., 2018; Wang et al., 2021). Moreover, this mutation partially rescued the lethality of mice lacking klotho, an anti-aging hormone whose deficiency causes accelerated aging, infertility, and premature death (Fernandez et al., 2018; Shi et al., 2020). Altogether, these studies show that an increment in autophagy levels in mammals can delay aging and age-related alterations and constitute a proof of concept enlightening the major focus of Beth Levine’s lab: the potential of autophagy stimulation in counteracting different pathological disorders.

### 2.2 Autophagy in Development

The embryonic development of *C. elegans* follows a well-known cell lineage from a 1-cell fertilized egg to a hatched larva, and therefore is especially useful for studying developmental cell death occurring during pattern formation in multicellular organisms. Huang et al. studied whether autophagy plays a role in the clearance of apoptotic cell corpses in the worm (Huang et al., 2013), and found that mutations in a series of autophagy genes caused increased numbers of these apoptotic corpses during embryogenesis, indicating that their efficient removal requires the autophagy machinery. Time-lapse analyses demonstrated that the increased corpses are likely due to their maintenance, with the genetic deletion mutant bec-1(ok691) having the most severe phenotype, indicating defects in the engulfment and degradation of corpses by the neighboring cells. In addition, rescue experiments showed that bec-1 is only required in the engulfing cells for the removal of apoptotic corpses. Further genetic epistasis analyses showed that bec-1 likely functions in parallel of known engulfment pathways ced-1/6/7 and ced-2/5/12 to promote the clearance of apoptotic corpses. Thus, it is possible that bec-1 mutation causes phagosome processing defects and impaired digestion of apoptotic corpses (Martinez et al., 2011), thus enhancing the clearance defects of the engulfment defective mutants.

### 2.3 TLR9-BECN1 Regulation of Energy Homeostasis

As a crucial catabolic pathway, autophagy activation in response to energy stresses denotes an important mechanism for maintaining cellular energy and nutrient homeostasis (Kroemer et al., 2010). It is well established that activation of AMP-activated protein kinase (AMPK), the master regulator of energy metabolism during energy stresses, promotes autophagy through various mechanisms including activating Class III PI3K complexes directly and indirectly (Galluzzi et al., 2014; Tamargo-Gomez and Marino, 2018). Works from the Levine lab further revealed the complexity of the relationship between AMPK signaling and autophagy pathways by showing that BECN1 and Class III PI3K complexes regulate AMPK activation during energy stresses such as exercise and glucose starvation (He et al., 2012; Liu et al., 2020). Specifically, it was first found that the dissociation of BECN1 from its inhibitory molecule, BCL2, during exercise is essential for the effective AMPK activation in skeletal muscle (He et al., 2012). It was further elucidated that after BECN1 dissociates from BCL2 during exercise, an innate immune receptor, Toll-like Receptor 9 (TLR9), recruits BECN1 and facilitates the assembly of the Class III PI3K-C2 at the endolysosome (Liu et al., 2020). In addition, it was shown that Class III PI3K-C2 is required for energy stress-induced AMPK activation (Liu et al., 2020). The loss of TLR9 in mice skeletal muscles results in impaired exercise-induced Class III PI3K-C2 assembly, AMPK activation, and subsequent glucose uptake. Thus, a novel TLR9-Class III PI3K C2-AMPK signaling axis that controls skeletal muscle energy metabolism during exercise was proposed, further illustrating the function of BECN1 in modulating metabolic adaptation in response to energy stresses through regulating the AMPK signaling pathway and providing an additional mechanism of how autophagy or autophagy proteins maintain cellular homeostasis during physiological and pathological stresses.

### 2.4 Autophagy and Cell Quiescence in Response to Nitrogen Starvation

The budding yeast *Saccharomyces cerevisiae* is a convenient model system to get a deeper understanding of the autophagic mechanism underlying nitrogen starvation, a robust autophagy inducer. Nutrient levels are important factors regulating the growing status of cells, and under nutrient deprivation, eukaryotic cells can exit the regular proliferation cycle and enter a quiescent stage G0 through G1. Given the critical roles of autophagy in regulating different pathways under starvation, it is possible that autophagy could also be critical in quiescence entry. Cell cycle profiles from control and yeasts with null mutations in different essential autophagy genes maintained in both normal growth or nitrogen starvation media were examined using imaging and flow cytometry. While all strains proliferated equally well and showed a normal cell cycle profile in a normal growth medium, yeasts that were deficient in autophagy failed to arrest in G1/G0 upon nitrogen starvation. Interestingly, those yeasts still showed quiescence-specific phenotypes such as an increase in Snf1 phosphorylation, trehalose, and glycogen levels. They failed to enter quiescence through G1 but were arrested at the telophase with a 2N DNA content. This study indicated that autophagy genes play essential roles for cell progression during nitrogen starvation in budding yeasts (An et al., 2014). Later reports in cells from higher eukaryotes also confirmed these findings, proving that autophagy genes are critical in proper quiescence entry through G1.

### 2.5 The Role of Autophagy in Cardiac Morphogenesis

Zebrafish (*Danio rerio*) has been widely used as a model for development and diseases. It is comparable with mammals in many aspects, although it is more efficient as a model system because of its fast embryonic development, tractability due to its transparency, and manageability for genetic studies (Dodd et al., 2000). The heart is one of the organs that have been well-studied in zebrafish. Unlike mammals, cardiac function in zebrafish embryos is dispensable for several days during early development. And embryos can uptake oxygen directly from their skin, which enables the fish to survive for several days without proper cardiac function (Pelster and Burggren, 1996).

Active autophagy was observed in multiple tissues during early embryonic development, and therefore the role of autophagy during embryonic development was investigated. Knockdown of key autophagy components such as atg5, beclin 1, and atg7 using morpholinos resulted in systemic developmental defects in the brain, body axis, and heart. In addition, autophagy-deficient zebrafish are defective in cardiac looping, abnormal chamber morphology, and aberrant expression of cardiac valve markers, which might be caused by abnormal regulation of cardiac patterning gene expression program. In line with this, Atg5-deficient mice displayed cardiac defects, including abnormal chamber septation. These results indicate that autophagy is necessary for normal cardiac morphogenic and gene expression programs during vertebrate development (Lee et al., 2014).

### 2.6 BECN1, Na^+^, K^+^-ATPase, and Autotic Cell Death

Even though autophagy is a pro-survival pathway aimed to maintain cellular homeostasis, it may also lead to cell demise under specific circumstances. Thus, it is important to understand how this process can switch from being beneficial to acting as a detrimental cellular response, as well as identify all the proteins implicated in its regulation. “Autophagic cell death” is a broad term applied to every cell demise that shows an accumulation of autophagic vacuoles and/or markers and that is dependent on autophagy-related genes, being blocked by the genetic or pharmacological inhibition of the route (Galluzzi et al., 2018). However, Beth Levine’s lab was able to identify a very specific type of autophagic cell death, termed “autosis,” that is also dependent on Na^+^,K^+^-ATPase (Liu et al., 2013).

Autosis, which can be repressed by cardiac glycosides (natural inhibitors of Na^+^,K^+^-ATPase), shows distinct ultrastructural features along with the presence of autophagic vacuoles, including increased substrate adhesion or the separation of the nuclear membranes, resulting in the expansion of the perinuclear space (Liu et al., 2013). Moreover, it has been detected in pathological conditions, from ischemic injuries in rats and mice to livers of patients with severe anorexia nervosa (Liu et al., 2013; Kheloufi et al., 2015; Fernandez et al., 2020), confirming the clinical relevance of this intriguing type of autophagic cell death.

Beth Levine’s work also unveiled that BECN1 plays a surprising role in autosis, as it directly interacts with Na^+^,K^+^-ATPase under autophagy- and autosis-inducing conditions like starvation, exercise, or ischemia (Fernandez et al., 2020). This unexpected association, which can be localized at different subcellular structures that include uncommon locations for the pump (like the mitochondrial or the nuclear membranes), can also be disrupted by cardiac glycosides, including those endogenously released by the organism. This crosstalk between two molecules oppositely involved in cellular energy balance may be crucial when defining cell fate under different conditions and could explain how autophagy can act either as a protective or a harmful process for the cell.

### 2.7 Exercise-Induced Autophagy

Work in the Levine’s lab also focused on the understanding of how physical exercise-induced autophagy mediates metabolic benefits. The research revealed for the first time that exercise potently induces autophagy, and autophagy activation by exercise is an essential underlying mechanism of exercise-mediated metabolic benefits. It was found that exercise-induced autophagy is regulated by BCL2, providing the first evidence that BCL2 functions as an autophagy regulator *in vivo*. Knock-in mutant mice containing mutations in three BCL2 phosphorylation sites important for exercise-induced autophagy were incapable of induce autophagy when exercising, and have decreased endurance and impaired glucose metabolism during acute exercise. More importantly, these mice are more susceptible to high-fat diet (HFD)-induced obesity, and fail to show protection from HFD-induced diabetes (and other metabolic improvements) by chronic exercise as observed in wild-type mice. These findings unveiled the therapeutic potential for autophagy activation in protecting against metabolic diseases, and laid the foundation for subsequent expanding studies on novel functions of autophagy during physical exercise. In addition, given the versatile protective roles of autophagy in diseases other than diabetes, including neuronal disorders, cancer and cardiovascular diseases, these findings raised the possibility that autophagy activation may contribute mechanistically to the beneficial effects of exercise on a broader spectrum of health problems (He et al., 2012).

### 2.8 The Discovery of BECN2

Besides in-depth studies on BECN1, an important genetic link between autophagy defects and metabolic diseases was found through a new autophagy gene, BECN2, and highlighted the functional and mechanistic diversity of BECN1 family members in autophagy, endolysosomal trafficking and metabolism. Specifically, it was found that BECN2 mediates agonist-induced lysosomal degradation of G protein-coupled receptors (GPCRs) by binding to the adaptor protein GASP1 (GPCR-associated sorting protein 1), and genetically dissociated this function of BECN2 from its role in autophagy by a substitution mutation in BECN2 that blocks its interaction with GASP1. Using knockout mouse models as genetic tools, the Levine lab also described that homozygous knockout of BECN2 in mice is embryonic lethal, and monoallelic loss of BECN2 results in metabolic dysregulation (including elevated food intake, obesity and insulin resistance) partially due to increased levels of a brain GPCR cannabinoid 1 receptor. Because the genetic locus of BECN2 is also linked to diabetic related traits in multiple ethnic groups in human, BECN2 likely represents an important genetic determinant of human obesity and diabetes. Thus, these findings represent a major advance in understanding the genetic mechanism that links autophagy to lysosomal downregulation of cell surface receptors and metabolic signaling. It opens up research directions to elucidate autophagy genes as a new class of direct regulators of GPCR signaling (He et al., 2013).

## 3 Autophagy in Cell Growth Regulation and Tumorigenesis

The role of autophagy in tumorigenesis is complex. On one hand, autophagy acts as a tumor suppressor; however, on the other hand, autophagy can also benefit tumor cells. Although autophagy prevents tumor initiation, it is thought to act as a pro-survival mechanism in established tumors by providing nutrients into tumor cells under metabolic stress. In addition, autophagy may have different functions in the context of different tumor mutational statuses.

BECN1 was initially identified as both an essential autophagy protein and a haploinsufficient tumor suppressor. The latter function of BECN1 is supported by several genetic evidence, including but not limited to 1) Single allele deletions are frequently seen in breast and ovarian cancers (Aita et al., 1999); 2) Heterozygous deletions of BECN1 in mice lead to increased incidence of breast and other spontaneous tumors (Qu et al., 2003; Yue et al., 2003; Cicchini et al., 2014); and 3) Reduced BECN1 mRNA expression is associated with poor prognosis in breast cancer (Tang et al., 2015). In addition to being genetically linked to tumors, BECN1 has established a closer association with cancer by mediating autophagy (Vega-Rubin-de-Celis, 2019). Although the specific mechanism whereby BECN1 inhibits tumor growth is not known, there is evidence suggesting that BECN1 modulates the subcellular localization of E-cadherin, highlighting a potential crosstalk between both proteins (Wijshake et al., 2021). However, the function of autophagy in cancer biology remains unclear and context-dependent, as demonstrated by the fact that autophagy can inhibit and promote tumor growth depending on the cancer subtype and tumor development stage. Therefore, a better understanding of the mechanisms by which autophagy is involved in tumorigenesis would be beneficial in developing new strategies to target autophagy in tumor prevention or cancer treatment. Here, we summarize some of the cancer-related findings arising from the Levine lab.

### 3.1 EGFR

EGFR tyrosine kinases (TK) are overexpressed or mutationally activated in a wide range of human cancers, including NSCLC (non-small cell lung cancer). Since active EGFR mutants are sensitive to small-molecule tyrosine kinase inhibitors (TKI), a subset of NSCLC driven by active EGFR mutants is usually treated with TKI erlotinib and gefitinib. Several studies have reported the relationship between EGFR signaling and autophagy during the development of NSCLC and TKI therapy. The downstream targets of EGFR, including PI3K, AKT, and mTOR, are well-recognized autophagy inhibitory molecules; therefore, autophagy is often induced in NSCLC during TKI therapy (Pao and Chmielecki, 2010). However, the function of autophagy is not clear in the TKI treatment, being postulated to exert either a cytoprotective or a cytotoxic role (Gorzalczany et al., 2011; Han et al., 2011).

Wei et al. (2013) demonstrated that when activated and internalized by endocytosis, EGFR binds and phosphorylates BECN1 at three tyrosine residues (Y229, Y233, and Y352), leading to stronger binding of BECN1 to the negative-regulatory proteins BCL2 and Rubicon, remodeling of the Class III PI3K complexes, decreased VPS34 kinase activity, and downregulation of autophagy. Treatment of TKI-sensitive NSCLC cells with TKI disrupted BECN1 phosphorylation and its interaction with Rubicon and BCL2, restored VPS34 activity, and triggered autophagy. Notably, this mechanism of autophagy regulation is independent of mTORC1 (mammalian target of rapamycin complex 1) activation, a well-recognized mechanism of autophagy downregulation (Fantin and Abraham, 2013).

Tyrosine phosphorylation by EGFR enhanced BECN1 homodimerization, making BECN1 more favorable for Rubicon binding and less favored for VPS34 binding, thereby inhibiting BECN1-associated VPS34 activity and suppressing autophagy. Substitution of these residues with phosphomimetic glutamic acid (E; BECN1-EEE mutant) produced a significant dominant-negative effect. Overexpression of BECN1-EEE in NSCLC cells inhibits VPS34 activation and autophagic flux upon TKI treatment, supporting the hypothesis that TKI response depends on BECN1 dephosphorylation (Wei et al., 2013). In tumor xenograft studies, tumors of NSCLC cells expressing BECN1-EEE exhibit lower basal autophagy and higher levels of apoptosis but, surprisingly, grew much faster than cells expressing the BECN1-wild-type (BECN1-WT) or the BECN1-AAA mutant (where phosphotyrosine sites were substituted with the non-phosphorylatable alanine, A). Further analysis identified a higher proliferation rate, an increased variance of nuclear DNA intensity, perimeter and area, and more entotic cells in BECN1-EEE tumors, which may compensate for the increased cell death associated with autophagy inhibition and contribute to the rapid growth of BECN1-EEE tumors. In addition, BECN1-EEE NSCLC xenografts were poorly differentiated, exhibiting a phenotype similar to the more aggressive squamous lung cancer, and promotes de-differentiation of NSCLC xenograft from TTF-1 (homeodomain protein highly expressed in lung adenocarcinoma) positive adenocarcinomas to TTF-1 negative tumors with squamous differentiation features. Established BECN1-EEE tumors were also significantly resistant to TKI therapy. Taken together these results suggest that the impaired autophagic response to EGFR inhibition contributes to TKI resistance (Wei et al., 2013).

### 3.2 HER2

Breast cancer is one of the most commonly diagnosed cancers worldwide. About 20% of breast cancers have amplifications in HER2 (ERBB2), a member of the EGFR family of RTKs, and this correlates with a more aggressive disease. In addition, some breast, bladder, and lung cancer also show mutations in HER2 at very low frequency. Targeted therapies with HER2 inhibitors are currently applied in clinical practice, including humanized monoclonal antibodies against the HER2 extracellular domain (Trastuzumab) and small-molecule ATP-competitive tyrosine kinase inhibitors (Lapatinib, Neratinib). However, such therapies produce adverse side effects, and most tumors develop resistance over time. Therefore, there is a need for finding more efficient alternative treatments, and autophagy was explored as a targetable pathway potentially implicated in the development of these cancers.

Using multiple HER2+ breast cancer cell lines, Levine lab showed that HER2 and BECN1 interact, and that such interaction is dependent on the HER2 activity, as a kinase-dead mutant of HER2 failed to co-immunoprecipitate with BECN1 (Vega-Rubin-de-Celis et al., 2018). In addition, HER2 overexpression (both WT and a kinase-active mutant, KA) inhibits autophagy. Interestingly, only HER2-KA mutant phosphorylates BECN1, suggesting that they may inhibit autophagy through different mechanisms.

Transgenic mice expressing HER2 into the mammary gland crossed with mice with elevated systemic autophagy (Fernandez et al., 2018) do not develop tumors, indicating that increased autophagy blocked HER2 tumorigenesis *in vivo*. Indeed, MEFs derived from Becn1^F121A^ mice overcame the HER2 inhibitory effect on autophagy.

In addition, treatment of HER2+ human breast cancer xenografts with the Tat-BECN1 autophagy-inducing peptide induced autophagy *in vivo* within the tumor, compromised the HER2/BECN1 interaction, induced a unique transcriptional profile and, most importantly, greatly inhibited tumor growth (Vega-Rubin-de-Celis et al., 2018; Vega-Rubin-de-Celis et al., 2020).

### 3.3 AKT

The PI3K-AKT signaling pathway, which is activated in many cancers, was also investigated, as it emerged as a potential regulatory node, especially since AKT had already been implicated in the regulation of cell survival through the modulation of the BH3-domain-containing protein, BAD (Datta et al., 1997). Using phosphospecific antibodies and phosphomotif mutants, we found that AKT could phosphorylate BECN1 on S234 and S295. The phosphorylation of BECN1 by AKT generates 14-3-3 binding sites, which sequesters BECN1 to intermediate filaments thereby inhibiting its interaction with VPS34, associated Class III PI3K activity, and autophagosome formation. Notably, mutation of the BECN1 AKT phosphomotifs to alanine generated a mutant that could partially inhibit the transforming activity of a constitutively activated, oncogenic AKT *in vitro* and in xenografts (Wang et al., 2012). In conjunction with DAPK (Zalckvar et al., 2009), AKT was amongst the first kinases identified to regulate BECN1 activity. However, additional studies ultimately revealed that BECN1 functions as a central regulatory hub for the modulation of autophagy and cell survival, and was also regulated by multiple other kinases implicated in tumorigenesis (Vega-Rubin-de-Celis, 2019).

### 3.4 MK2/3

Phosphorylation is the most extensively studied post-translational modification of BECN1 (Levine et al., 2015; Hill et al., 2019). Using mass spectrometry, S90 was identified as a major phosphorylation site of BECN1 upon starvation, and a kinase screen identified two mitogen-activated protein kinase (MAPK)-activated protein kinases, MAPKAPK2 (MK2) and MAPKAPK3 (MK3) as the kinases phosphorylating BECN1-S90 (Wei et al., 2015). This phosphorylation event positively regulates autophagy, as suggested by multiple lanes of evidence: 1) MK2/MK3 double knockout, 2) blocking MK2/MK3 activity with dominant-negative MK2, or 3) replacing wild-type (WT) BECN1 with a non-phosphorylated form S90A, prevented the initiation of autophagy during starvation. In contrast, overexpressing active MK2 or the phospho-mimetic BECN1-S90E induces autophagy even in the presence of sufficient nutrients. Thus, BECN1 S90 phosphorylation promotes autophagy by enhancing the VPS34 lipid kinase activity of Class III PI3K complex 1 (Wei et al., 2015), and BCL2 binding may inhibit BECN1 S90 phosphorylation by MK2.

This finding is also relevant for cancer research, as phosphorylation of S90 (and the corresponding autophagic function) is also required for tumor suppression by BECN1. Compared with its WT counterpart, expression of phosphorylation- and autophagy-deficient S90A BECN1 failed to inhibit MCF-7 xenograft growth in immunodeficient nude mice (Wei et al., 2015).

MK2/MK3 is activated during amino acid starvation by the well-characterized upstream MAPK, p38α (Trempolec et al., 2013). An earlier work by Wei et al. reported that under starvation, BCL2 is phosphorylated by c-Jun N-terminal protein kinase 1 (JNK1), another MAPK parallel to p38. JNK1 phosphorylation of BCL2 at multiple sites is required to release BECN1 during starvation to initiate autophagy (Wei et al., 2008). Therefore, Wei et al. discovered a central regulatory loop that underlines starvation-induced activation of autophagy, containing two distinct arms of the MAPK signaling pathway that act in parallel to activate the autophagic function of BECN1 in response to starvation. JNK1-mediated BCL2 multisite phosphorylation, which leads to disruption of BCL2/BECN1 interaction, is a prerequisite step for subsequent MK2/MK3-dependent BECN1 S90 phosphorylation and BECN1-dependent autophagy. These two arms act synergistically to mediate autophagy at the level of BECN1 S90 phosphorylation, emphasizing the critical importance of the interaction between MAPK signaling pathway and BECN1 in starvation-induced autophagy. More broadly, these findings suggest that activation of autophagy is intricately coordinated with other MAPK signaling in response to stress (Wei et al., 2008; Wei et al., 2015).

### 3.5 Tumorigenesis Studies in Zebrafish Models

Zebrafish has become a popular model for the study of cancer as these animals develop many kinds of tumors that share similar morphology and comparable pathways with those in humans. However, there are also several differences to mammalian tumorigenesis including a low tumor incidence compared to the mouse models and a different tumor spectrum. Still, there are also advantages of using zebrafish as a model system, including its suitability for genetic and chemical screening, and for *in vivo* tumor progression imaging.

Using the Tol2 transposon system to generate genomic insertions of the mitf:atg5^K130R^ transgene, expressing a dominant-negative ATG5^K103R^ (dnATG5), stable transgenics Tg(mitfa:dnatg5) were generated, raised to adulthood, and monitored for tumor onset. In contrast with previous reports (Tian et al., 2015), stable transgenic fishes did not develop tumors. Since the inhibition of autophagy alone might be insufficient to develop tumors in zebrafish, the effect of autophagy defects in the tp53 mutant background was tested. Interestingly, in such background (tp53^M214K^), an impaired autophagy fish line was prone to develop tumors, including malignant peripheral nerve sheath tumors (MPNSTs). Expression of dnATG5 significantly increased tumor incidence and decreased latency in p53-deficient fish. Nearly 40% of autophagy defect transgenic fish Tg(mitfa:dnatg5);tp53^M214K/+^ developed tumors with a median age of onset of 17 months compared to tp53^M214K^ heterozygotes exhibiting 17% of incidence and longer latency, with a median age of onset of 20 months. In addition, tumors arising in Tg(mitfa:dnatg5);tp53^M214K/+^ exhibited increased generation of double-strand DNA breaks and displayed loss of heterozygosity (LOH) for p53, suggesting a possible role for autophagy in tumor suppression by regulating genomic stability and LOH of tumor suppressor genes (Lee et al., 2016).

## 4 Selective Autophagy Factors

In addition to describing BECN1 and its role on autophagy, Beth Levine contributed to the identification of mammalian proteins required for selective autophagy, as well as the regulation of key autophagic processes. Autophagy was believed to be a non-selective degradative process required to generate energy and maintain homeostasis when cells are grown in low nutrient conditions (starvation). However, autophagy also functions to recognize and target for degradation specific cargoes in a highly selective manner, in a process known as selective autophagy (Gatica et al., 2018). Selective autophagy pathways require the same core autophagy machinery used by non-selective autophagy and involve additional components known as selective autophagy factors. Such factors are responsible to identify and deliver specific substrates to the autophagic machinery (Stolz et al., 2014). Depending on the substrate targeted by autophagy, selective autophagy can be divided into a variety of subtypes including xenophagy (intracellular pathogens) (Deretic, 2011), virophagy (viruses) (Dong and Levine, 2013), pexophagy (peroxisomes) (Germain and Kim, 2020), mitophagy (mitochondria) (Onishi et al., 2021), amongst others (Gatica et al., 2018). The identification of determinants of selective autophagy and the mechanisms by which they work to target substrates for autophagic degradation is crucial to understanding how autophagy works in several physiological processes and may contribute to the development of new therapies against a diverse set of diseases, including cancer and infections.

### 4.1 Smurf1

Beth Levine was a pioneer in the use of a high-throughput genetic approach to identify mammalian genes essential to mediate distinct types of selective autophagy (Orvedahl et al., 2011). By using a genome-wide, image-based screen and bioinformatic analyses, Levine’s lab identified over 90 genes specifically required for both virophagy and mitophagy (and not required for regulation of non-selective autophagy). To further explore the biological role of these genes in selective autophagy, studies were carried out using cells or mice defective for selected individual genes. One of the genes identified encodes the protein E3 ubiquitin ligase Smurf1, which is important for targeting for ubiquitination and degradation of protein substrates related to cell growth, cell migration, bone formation, and cancer progression (Cao and Zhang, 2013). Beth Levine’s lab showed that cells derived from mice deficient for Smurf1 presented defective targeting of viral capsids to autophagosomes and accumulated damaged mitochondria in the brain, heart, and liver (Orvedahl et al., 2011), demonstrating the involvement of Smurf1 in selective autophagy of viruses and damaged mitochondria. In addition to virophagy and mitophagy, Smurf1 was demonstrated to be required for xenophagic clearance of the intracellular bacteria *Mycobacterium tuberculosis*, the causative agent of tuberculosis in humans (Franco et al., 2017). Smurf1-deficient macrophages were more permissive to *M. tuberculosis* replication and Smurf1-deficient mice succumb more quickly than wild-type mice to tuberculosis, due to impaired delivery of *M. tuberculosis*-containing phagosomes to autophagosomes (Franco et al., 2017). These results point to an essential role for Smurf1 in mediating host resistance against viral and bacterial infections.

### 4.2 Fanconi Anemia Genes

Among the many hits from the genetic screen for selective autophagy were FANCC, FANCF, and FANCL, which when mutated causes a rare disease called Fanconi Anemia (FA). FA is caused by autosomal homozygous germline mutations (biallelic) in any of the 22 FANC genes from FANCA to FANCW (although FANCB is on the X chromosome and thus monoallelic mutations in males can result in disease). These mutations can lead to a range of clinical outcomes including congenital defects, bone marrow failure, and cancer predisposition. The main function of FA genes is to repair Inter-strand Cross Links (ICL) caused by exogenous insults or by endogenous reactive oxygen species and aldehydes (Niraj et al., 2019). Apart from DNA repair functions, some FA proteins also have cytoprotective functions (Pang et al., 2001). Beth Levine showed that the gene FANCC, though not essential for starvation-induced general macroautophagy, is involved in regulating mitophagy, virophagy, and suppresses inflammasome hyperactivation by removing damaged mitochondria and associated mitochondrial reactive oxygen species (Sumpter et al., 2016). The focus on FANCC was justified by its role in cytokine hypersensitivity, which is independent of its role in nuclear DNA damage repair (Pang et al., 2001). They further found that the role of FA genes in mitophagy is independent of their roles in DNA damage repair by showing that the naturally occurring hypo-morphic mutant of FANCC, FANCC c.67delG (which encodes a shorter protein lacking the N-terminal 55 amino acids of FANCC) fully complements the cytoprotective function involving mitophagy, but not the genomic DNA repair function. Mitochondria are an important source of endogenous ROS generation, which causes oxidative stress and damage to DNA, lipids, and proteins. Failure to clear damaged mitochondria can lead to disease and may therefore be a factor in Fanconi Anemia. They also showed that FANCC is required for virophagy of two genetically distinct neurotropic viruses; Sindbis virus (a positive single-stranded RNA-based genome) and Herpes Simplex Virus Type I (a double-stranded DNA-based virus) (Sumpter et al., 2016). Thus, they showed that FANCC is involved in two different types of selective autophagy. This discovery of the role of FA genes in selective autophagy furthers our understanding of the mechanisms by which mutations in FA genes lead to human diseases, including childhood cancer, and also helps in developing therapeutic strategies to treat these patients.

### 4.3 PEX13

Another hit found in Beth Levine’s genetic screen was the gene that codes for PEX13, a peroxisomal protein required for peroxisome formation (Fujiki et al., 2014). Mutations in PEX13 gene are related to the development of Zellweger syndrome spectrum (ZSS) disorders (Liu et al., 1999). Levine’s lab showed that ZSS-associated PEX13 mutants have defective mitophagy and PEX13-mediated mitophagy may be related to ZSS pathogenesis (Orvedahl et al., 2011; Lee et al., 2017).

### 4.4 PHB2

Studies in the field of mitophagy largely focused on the molecular events that direct the recognition of the damaged mitochondria at the outer mitochondrial membrane (OMM), including the stabilization/activation of PINK1, the recruitment of Parkin, the ubiquitination of mitochondrial outer membrane proteins, and the recruitment of ubiquitin-binding adaptors (Bingol et al., 2014; Pickrell and Youle, 2015; Yamaguchi et al., 2016). The inner mitochondria is physically separated from the cytosol and was not thought to mediate cargo recognition during mitophagy. Previous studies reported that the OMM undergoes proteasome-dependent rupture during Parkin-mediated mitophagy (Chan et al., 2011; Yoshii et al., 2011), indicating that phagophore may directly reach mitochondria intermembrane spaces (IMS) and inner mitochondrial membrane (IMM) during mitophagy. However, the exact mechanistic link between proteasome-dependent OMM rupture and mitophagy is not known. In addition, earlier identified mitophagy receptors such as FUNDC1, BNIP3 and NIX are all OMM proteins, and can interact directly with LC3 through their LC3-interacting region (LIR) motifs to facilitate mitophagy (Green and Levine, 2014). Although the PINK1-Parkin pathway is not always required for mitophagy, it is a critical amplifying mechanism that renders mitophagy more efficiently (Villa et al., 2018). The above model of mitophagy, in which the OMM receptors serve as primary targets for autophagosome recognition and engulfment, is not fully reconciled with all mitophagy-inducing signals (such as depolarization and the presence of paternal mtDNA) being derived from within the mitochondria. Thus, other mechanisms may exist to target and ensure the destruction of the inner mitochondrial compartment.

Wei et al. employed tandem affinity purification of the LC3-II-interacting complex in Parkin-overexpressing Hela cells undergoing mitophagy to investigate mitophagy-regulated LC3 interactome in mammalian cells and identified a mitochondrial inner membrane protein, prohibitin 2 (PHB2), as a novel mitophagy receptor (Wei et al., 2017). Co-immunoprecipitation and *in vitro* binding experiments showed that LC3-II directly binds to PHB2. When HeLa-Parkin and MEFs-Parkin cells were treated with OA (a combination of oligomycin and antimycin), depletion of PHB2 resulted in defective clearance of depolarized mitochondria, suggesting that PHB2 is required for Parkin-dependent mitophagy (Wei et al., 2017). PHB2 mediates autophagic clearance of mitochondria via its direct interaction with LC3 through a canonical LIR motif upon mitochondrial depolarization.

Given that PHB2 and LC3 are topologically separated by the OMM, Wei et al. hypothesized that the integrity of the OMM must be disrupted to allow PHB2/LC3 interaction during mitophagy. Indeed, Parkin has been suggested to be involved in rupturing OMM during mitophagy by tagging OMM proteins with ubiquitination for proteasomal degradation (Chan et al., 2011; Yoshii et al., 2011). Transmission electron microscopy demonstrated that OMM rupture was only observed in mitochondria purified from OA-treated HeLa-Parkin but not HeLa-vector cells. In addition, the rupture was entirely prevented by treatment with a potent proteasome inhibitor, epoxomicin. Consistently, in Parkin-expressing cells treated with OA, the epoxomicin treatment also prevented colocalization and interaction of PHB2/LC3-II and mitochondrial clearance. By using super-resolution microscopy and immuno-electron microscopy, Wei et al. obtained direct evidence supporting the model of IMM receptor-mediated mitophagy, identifying LC3-decorated phagophore crossing the OMM rupture cleft and making direct contact with PHB2 on the IMM. However, in PHB2-knockdown or epoxomycin-treated cells, the phagophore could only approach but not contact the mitochondria, and therefore could not mediate mitochondria encapsulation by autophagosomes (Wei et al., 2017). Thus, proteasome-dependent rupture of the OMM is necessary for PHB2/LC3 interaction and mitophagy, indicating that the topologically exposed PHB2 is a molecular signature of the ruptured mitochondrial membrane sensed by selective autophagy.

This study not only pinpointed a novel mechanism of mitophagy but also suggested that cargo recognition at the IMM during mitophagy serves as an additional layer of regulation that ensures selectivity and efficiency of mitochondrial clearance.

Overall, Dr. Levine’s lab filled a gap in the field of selective autophagy identifying new autophagy factors and describing the molecular mechanisms by which they promote selective elimination of virus and bacteria, in addition to comprehending the pathogeneses of genetic diseases.

## 5 Autophagy in Neurodegeneration

Autophagy is an important mechanism for the clearance of aggregated proteins. Thus, its dysregulation has been linked to neurodegenerative disorders like Alzheimer’s disease, Parkinson’s disease, or Huntington’s disease. In *C. elegans*, Jia et al. (2007) transgenically express polyQ repeats and find that RNAi knock-down of autophagy genes bec-1, atg-7, and atg-18 accelerate the formation of polyQ aggregates (Jia et al., 2007). Autophagy deficiency also enhances motility reduction and neurodegeneration when polyQ is expressed in muscle cells or neurons, respectively. Ultrastructural analysis shows that autophagy is induced by polyQ expression. However, no autophagosomes are found around the large polyQ aggregates, suggesting that autophagy may only target polyQ before the formation of aggregates. These results demonstrate a role for autophagy in suppressing the accumulation of polyQ aggregates and providing protection in the *C. elegans* model of polyQ expansion disease.

The mouse model with increased autophagy levels that was generated in Beth Levine’s lab (Becn1 F121A knock-in mice) has been also a useful tool to assess the protective role of autophagy in neurodegeneration. For instance, this mutation enhances the autophagic pathway in the brain, boosting the clearance of amyloid oligomers and improving cognition in an animal model of Alzheimer’s disease (Rocchi et al., 2017). Moreover, the increased autophagy of these mice prevented neural stem cells exhaustion during aging, promoting neurogenesis in old animals (Wang et al., 2021), showing that autophagy can also protect from neurodegeneration in mammals.

## 6 Autophagy in Infection

In *C. elegans* the daf-2 pathway also controls pathogen resistance, and therefore Jia et al. (2009) examined whether autophagy provides a protective effect against infection by *Salmonella typhimurium* via the daf-2 pathway (Jia et al., 2009). In wild-type worms, *Salmonella* that enters the intestinal cells is detected inside autophagosomes/autolysosomes and is often found partially degraded. When bec-1 is knocked down, few or no autophagosomes are detected in the intestinal cells, and *Salmonella* replicates intracellularly to reduce survival. While daf-2(e1370) worms are resistant to *Salmonella* infection, RNAi depletion of autophagy genes bec-1 and lgg-1 reduced the survival of daf-2(e1370) worms, demonstrating that autophagy underlies the pathogen resistance conferred by a defective insulin-like signaling pathway.

More recently, using a genome-wide, image-based genetic screen, Levine’s lab identified that the endosomal protein sorting nexin 5 (SNX5) is a host factor required for autophagosome formation during virus-induced autophagy (but not for basal- or stress-induced autophagy) and for host resistance against viral infections (Dong et al., 2021). Disruption of SNX5 gene in cells or mice increased intracellular replication and susceptibility of several viruses including Sindbis virus, herpes simplex virus type 1, West Nile virus, and chikungunya virus (Dong et al., 2021). Mechanistically, SNX5 works on viral-induced autophagy by increasing autophagosomal formation at virion-containing endosomes (Dong et al., 2021).

## 7 Autophagy Modulators and Therapeutics

Given the pivotal roles of autophagy in cellular homeostasis, immunity, tumor suppression, metabolism, prevention of neurodegeneration, and lifespan extension, pharmacological augmentation of autophagy is thought to be an effective approach to treat human diseases or to promote longevity. Many autophagy-inducing agents are currently in clinical trials or use, but the effects of these agents are pleiotropic and the mechanism of actions are not specific to the autophagy pathways. Thus, the development of autophagy inducers that selectively target key regulatory steps of autophagy, rather than the upstream signaling pathways, is warranted.

### 7.1 Identification of Small Molecules That Selectively Block BECN1/BCL2 Interaction

A key checkpoint of autophagy regulation is the interaction between BCL2 and BECN1. BCL2 proteins negatively regulate autophagy and apoptosis via binding of the BH3 domain of BECN1 and pro-apoptotic proteins (Feng et al., 2007; Oberstein et al., 2007). A class of compound known as BH3 mimetics were shown to disrupt the interaction of BECN1 and BCL2 proteins to induce autophagy (Maiuri et al., 2007; Pedro et al., 2015) but these agents also induce apoptosis. Thus, pharmacological agents that selectively disrupt the binding of BECN1 and BCL2 are desirable. Biochemical studies indicated that there exist differences in the binding modalities of BCL2/BH3 domain interactions (Feng et al., 2007; Oberstein et al., 2007; Su et al., 2014), suggesting that selective pharmacological disruption of BECN1/BCL2 interaction is possible. To this end, the Levine’s lab developed high throughput assays (split-luciferase assay and AlphaLISA, proximity-based systems that measure protein-protein interaction) for BECN1/BCL2 and BAX/BCL2 interaction and performed a drug screen. Three compounds were identified to disrupt BECN1/BCL2 interaction and stimulate autophagy at the micromolar range (Chiang et al., 2018). At working concentrations, these molecules do not disrupt the binding of BCL2 to the BH3 domains of BIM and BAX (pro-apoptotic BCL2 family members) nor stimulate apoptotic cell death. Such selectivity indicated that these compounds may induce basal autophagy without sensitizing cells toward apoptosis. NMR chemical shift perturbation analysis suggested that these molecules may bind to BCL2 via distinct binding modes different from other BH3 mimetics, providing a mechanistic basis for their selectivity towards BECN1/BCL2 interaction.

Recent studies in mice demonstrated that the targeted mutation (F121A) of BECN1 that decreases its interaction with BCL2 results in elevated basal autophagy and is associated with the promotion of healthspan and longevity (Fernandez et al., 2018). BECN1 knock-in mutation also reduces amyloid oligomers and improves cognitive function in a mice model of Alzheimer’s disease (Rocchi et al., 2017), and reduces HER2-driven breast cancer tumorigenesis in mice (Vega-Rubin-de-Celis et al., 2018). These findings suggested that the pharmacological approach of promoting basal autophagy via the selective disruption of BECN1/BCL2 may be safe and have beneficial effects in mammals. Thus, the BECN1/BCL2 inhibitors identified in this study may serve as starting molecules for future structure-activity relationship (SAR) studies to develop more selective and effective autophagy-inducers, which can be used for the treatment of a variety of human diseases in which the promotion of autophagy is beneficial.

### 7.2 Identification of a Candidate Therapeutic Autophagy-Inducing Peptide

Deletion scanning analysis in BECN1 identified a region [BARA (β-α repeated, autophagy-specific) domain β-sheet 1 comprising amino acids 267-284, required for the autophagic function of BECN1, within the BECN1 evolutionarily conserved domain (ECD)], responsible for the BECN1 interaction with a negative regulator of autophagy HIV-1 Nef. It was hypothesized that such amino acids in BECN1 may be sufficient to induce autophagy, and a cell-permeable peptide was designed, Tat-BECN1, composed of the HIV-1 Tat protein transduction domain (PTD) attached to 18 amino acids derived from amino acids 267-284 of BECN1. Treatment with Tat-BECN1 peptide, but not a control peptide (Tat-scrambled), resulted in dose-dependent autophagy induction in multiple cell lines and primary MEFs (Shoji-Kawata et al., 2013).

GLIPR2/GAPR-1 (Golgi-associated plant pathogenesis-related protein 1), was identified as a protein that may mediate the autophagy-inducing effect of the Tat-BECN1 peptide. GLIPR2 is therefore a new BECN1 interactor, that associates with lipid rafts as the cytosolic leaflet of the Golgi membrane (Eberle et al., 2002). BECN1 binds to GLIPR2 via the BARA domain β-sheet 1 (Shoji-Kawata et al., 2013) and Class III PI3K-C1 complex (Zhao et al., 2021), and GLIPR2 deletion leads to increased autophagy levels *in vitro* and *in vivo* (Shoji-Kawata et al., 2013; Zhao et al., 2021). Knockdown of GLIPR2 increased numbers of autophagosomes in both Tat-scrambled and Tat-BECN1-treated cells, due to an increase in autophagic flux rather than a block in autophagosome maturation. It was determined that GLIPR2 may function to tether BECN1 to the Golgi apparatus (inactivating autophagy) and that Tat-BECN1 peptide may promote its release from the Golgi, resulting in enhanced early autophagosome formation. Taken together, these data indicate that GLIPR2 is a BECN1-interacting protein that negatively regulates autophagy. However, GLIPR2 deletion does not dampen BECN1 peptide-induced autophagy, hinting at the existence of other targets (Zhao et al., 2021). Indeed, BECN1 peptide directly enhances the lipid kinase activity of Class III PI3K-C1 and -C2 complexes (Chang et al., 2019; Zhao et al., 2021), suggesting that BECN1 itself is a target. The negative regulation of GLIPR2, HIV-1 Nef, Rubicon and the positive regulation of BECN1 peptide, on the lipid kinase activity of Class III PI3K complexes and on autophagy (Shoji-Kawata et al., 2013; Chang et al., 2019; Zhao et al., 2021) demonstrate the important regulatory role of BECN1 BARA domain β-sheet 1, which is supported by structural analyses of membrane association of Class III PI3K kinase complexes (Rostislavleva et al., 2015; Ohashi et al., 2020). BECN1 peptide derived from this region acts as a potent autophagy activator *in vitro* and *in vivo*, when fused with cell-penetrating peptide HIV Tat (Shoji-Kawata et al., 2013) or stapled by crosslinking (Peraro et al., 2017). Studies from Levine’s lab and others further confirmed that BECN1 peptide robustly induces autophagy in around 50 cell lines and primary cell cultures and over a dozen tissues. BECN1 peptide exhibits protective effects against viral infections (Shoji-Kawata et al., 2013; Gassen et al., 2019; Zhang et al., 2019; Kobayashi et al., 2020), bacterial infections (Shoji-Kawata et al., 2013; Miao et al., 2015; Franco et al., 2017; Nikouee et al., 2021), and endotoxemia and sepsis (Sun et al., 2018; Nikouee et al., 2021); clears damaged mitochondria and alleviates ischemia/reperfusion injuries in the heart (Xie et al., 2021) and kidney (Livingston et al., 2019); and reduces the growth of tumor xenografts (Wang et al., 2015; Ding et al., 2018; Song et al., 2018; Vega-Rubín-de-Celis et al., 2018; Zhou et al., 2019). BECN1 peptide improves the fertility and reproductive lifespan (Watanabe et al., 2020), enhances the formation of long-term spatial memory (Hylin et al., 2018), and rescues age-related memory decline (Glatigny et al., 2019; De Risi et al., 2020), without causing systemic toxicity. In addition, Levine’s lab was also able to demonstrate that prolonged treatment of Tat-BECN1 peptide induces a new type of autophagy-dependent, apoptosis and necrosis-independent cell death, autosis (Liu et al., 2013).

In summary, a functionally important region of BECN1 was identified, leading to the synthesis of a cell-permeable autophagy-inducing peptide. Specific autophagy-inducing agents such as Tat-BECN1 peptide may have the potential for the prevention and treatment of a broad range of human diseases.

## 8 A Little Personal Note From the Authors

**FIGURE 2:**
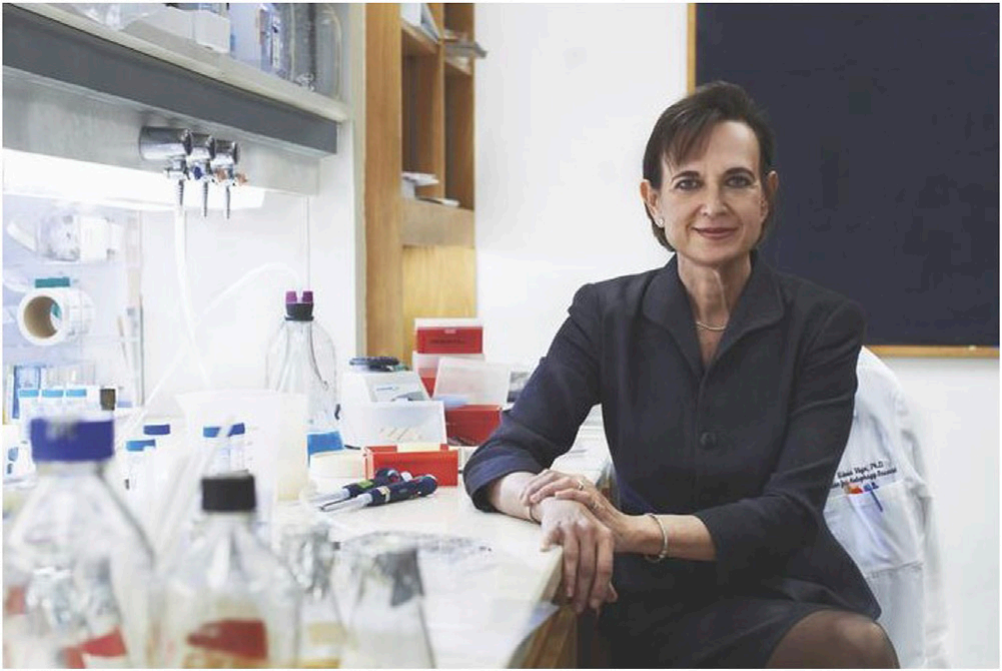
Our very own Beth Levine.

Zhenyi An: During the years in the Levine lab, I learned so much from Beth. Her passion for scientific research; her commitment to mentoring trainees; her collaborative spirit and her courage in overcoming obstacles are always there to inspire me. Beth sets a great role model for younger scientists. She will be remembered forever (Figure 2).

Wei-Chung Chiang: Beth is a caring and supportive mentor. Her passion and attitude toward science have a great influence on my mentoring style. I will continue to inspire my trainees with the Levine lab spirit. We miss her.

Álvaro F. Fernández: Beyond her invaluable scientific achievements, Beth Levine also stood out as an exceptional mentor and colleague, thanks to her limitless kindness, her contagious cheerfulness, her unwavering support, and her excellence. Always willing to guide, teach, collaborate, and help, she will deservedly be remembered as one of the pioneers and leaders of autophagy research.

Luis H. Franco: I would like to express my gratitude to Dr. Levine and her family for the incredible experience I had with her, both personally and professionally. She was very good at discussing and presenting her ideas, always in a clear and objective way. She knew how to choose very well the words, which were pronounced sweetly and at the same time secure. The example I will carry with me for the rest of my life is her professionalism and earnestly. She always treated us professionally and equally. During the 5 years I have been in her lab, she helped me to find my way into science. Because of all this and thanks to her, today I am part of one of the best Universities and Scientific Researcher Center in Brazil.

CongCong He: Beth was an incredible mentor and role model. She was both fearless and cautious in science. Because of this, the numerous scientific findings in her lab have stood the test of time and led many important new research directions on-going in the field.

Shu-Yi Huang: Dr. Levine was a great mentor in science and life. She was sharp and meticulous when doing science but was considerate and supportive toward people. She was a true leader–full of pioneering ideas and a deep passion for research. It was a privilege to have learned from her. I will always miss her elegant style.

Eunmyong Lee: “Remember, the important is quality of time, not quantity,” Dr. Beth Levine said at a seminar for women in science. On those days, I was almost exhausted and wondered if I was on the right track as a mother of a newborn. She said that a working mother is the best role model for her kids. She told us how she managed the work-life balance and encouraged us to be proud of ourselves as women scientists. She was a very supportive, dedicated, and inspiring mentor. To me, Dr. Beth Levine was not only an academic supervisor but also a role model as a great female scientist.

Yang Liu: I am so grateful to have spent 6 years in the Levine lab under the marvelous mentorship of Beth. Beth taught me not only about how to perform great research but also about the “try harder” and “give it your best” attitudes of living. We will carry on Beth’s legacy to make new discoveries in biomedical sciences as well as to thrive in our lives without regrets.

Salwa Sebti: Beth Levine will always be remembered as a pioneer of the autophagy field. I will also always remember her for her kind heart, her truthfulness and brilliant mind, and will cherish the memories of our long conversations.

Sanae Shoji-Kawata: I really respect Dr. Beth Levine as a researcher and a woman. I sometimes felt that she was my elder sister. I learned a lot of research-related things and life from her. In research, the discussions with her were always great lessons for me, which made my thinking in a more sophisticated way. In life, she had always encouraged me.

Shyam Sirasanagandla: I feel extremely privileged to have the opportunity of working under Dr. Beth Levine. I had gained so much insight and technical skills in her lab. Her intellect and vision had driven and inspired so many scientists not only from her own lab but also from around the world to come up with various important discoveries. Her contribution to the field of Autophagy and Science at large will always have a special place in the scientific world.

Silvia Vega-Rubín-de-Celis: Dr. Levine was always eager to kindly support people in her lab both professionally and personally. On the scientific side, she was always supportive to pursue my ideas. On the more personal side, as a mother in science, she was always the person to listen and willing to help in any possible way.

Yongjie Wei: As the longest-serving member of the Levine group, I have had the privilege of working with Beth for nearly 16 years and have been around for almost her entire career at UT Southwestern. Over the years, I have watched member after member joining the lab, complete their training and leave to embark on their next journey. I have witnessed Beth’s tireless and careful mentoring process with myself and each individual. Beth had a keen eye for the broad interests and scientific issues in the field, and I was constantly amazed by the brilliant ideas she came up with for each project. Meanwhile, she also put a lot of effort into little details like punctuation, italics, capital letters, etc., in our manuscripts. Over the years, she never skimped on her time and had several practice sessions with each lab member to give a presentation. After having my own group, I always subconsciously imitated and followed Beth’s way of training graduate students and managing the lab. With Beth’s departure, I have lost a mentor, a long-time colleague, a close friend, and sometimes a big sister whom I could count on in any difficulty. Beth, we will always miss you.

Richard Wang: Beth Levine was an outstanding science communicator. She had a gift for writing articles in a clear and convincing way. Her reviews on autophagy remain some of the most influential and highly cited in the field. Her passion for science helped to bring countless researchers to the field of autophagy.

Yuting Zhao: Beth was more than a postdoctoral mentor to me. She was a role model and a caring friend. I missed her so much especially when my work was published in late 2020 and when I started my own lab in 2021. We will continue the scientific journey that Beth began.
